# Supplementary material for: Performance of screening tools for cervical neoplasia among women in low- and middle-income countries: A systematic review and meta-analysis
Source: PLOS Glob Public Health. 2023 Feb 23;3(2):e0001598. doi: 10.1371/journal.pgph.0001598 (PMC10021669; doi:10.1371/journal.pgph.0001598)
Supplement: Attachment — Submitted filename: LetterstoReviewers.docx [file pgph.0001598.s007.docx]

Dear Editors and Reviewers,

We thank you for the positive and thoughtful review comments. We have responded to each of the comments in the ***TABLE*** below and have made the corresponding changes in the text of our revised manuscript. We have attached updated manuscript versions (a clean copy and a copy with changes tracked). We believe these changes have strengthened our paper and look forward to hearing back from the journal. To ensure a thorough, point-by-point response, we have responded to each of the comments in the table under the “Author Reply” column in the ***TABLE***. Thank you!

***TABLE. Response to Reviewers.***

| **Comments to Authors Category** | **Reviewer Responses** | **Author Reply** |
| --- | --- | --- |
| **1. Does this manuscript meet PLOS Global Public Health’s** [**publication criteria**](https://nam11.safelinks.protection.outlook.com/?url=https%3A%2F%2Fjournals.plos.org%2Fglobalpublichealth%2Fs%2Fcriteria-for-publication&data=05%7C01%7Ckara.suvada%40emory.edu%7C61d9f2161e6a46f250c708dac25e8ee8%7Ce004fb9cb0a4424fbcd0322606d5df38%7C0%7C0%7C638036008654662825%7CUnknown%7CTWFpbGZsb3d8eyJWIjoiMC4wLjAwMDAiLCJQIjoiV2luMzIiLCJBTiI6Ik1haWwiLCJXVCI6Mn0%3D%7C3000%7C%7C%7C&sdata=HuqgyB36GEz8240W9cQL4FPdfxZ24FR2sbM3uauWo7o%3D&reserved=0)**? Is the manuscript technically sound, and do the data support the conclusions? The manuscript must describe methodologically and ethically rigorous research with conclusions that are appropriately drawn based on the data presented.** | Reviewer #1: Yes | Thank you. |
|  | Reviewer #2: Yes |  |
|  | Reviewer #3: Yes |  |
| **2. Has the statistical analysis been performed appropriately and rigorously?** | Reviewer #1: Yes | Thank you. |
|  | Reviewer #2: Yes |  |
|  | Reviewer #3: Yes |  |
| **3. Have the authors made all data underlying the findings in their manuscript fully available (please refer to the Data Availability Statement at the start of the manuscript PDF file)?** | Reviewer #1: Yes | Thank you. |
|  | Reviewer #2: Yes |  |
|  | Reviewer #3: Yes |  |
| **4. Is the manuscript presented in an intelligible fashion and written in standard English?** | Reviewer #1: Yes | Thank you. |
|  | Reviewer #2: Yes |  |
|  | Reviewer #3: Yes |  |
| **5. Review Comments to the Author** | | |
| *Reviewer #1* | | |
| Thank you for the opportunity to review this manuscript on the performance of screening tools for cervical neoplasia among women in low and middle income countries. The manuscript is well written. | | Thank you for your thoughtful comments about our manuscript. |
| The test parameters for the screening tests would look very different in a high income country. The authors did not comment on this in the discussion. It is very important as decisions on a screening test should be made on results from the test in the environment in which it will be used. | | Thank you for this thoughtful comment. Per your comment, we searched in the literature for the use of methods other than Pap smear in high-income countries and there is almost no literature. We have added the following sentence to the discussion to emphasize your comment: *“Future studies should explore test performance of screening methods in expanded geographies, to include rural populations, and low-income countries, and high-income countries”.* |
| Lines 261-263 are a repeat of lines 259-261 | | The repeated sentence has been removed, thank you. |
| *Reviewer #2* | | |
| This study addresses an important research question and adds to the existing literature on the subject. I do have a few comments that in my opinion will strengthen this paper. | | Thank you for your positive and constructive feedback. |
| Whereas most readers understand the concepts of sensitivity and specificity, they may be less familiar with diagnostic odds ratio and the pooled receiver operating characteristic curve. It would be good define all of these measures (with equations if possible) early in the Methods section. | | Thank you for pointing this out. In line 182-185, we added definitions of the diagnostic odds ratio and pooled receiver operating characteristic curve. The sentences are as follows: *“The DOR is the odds of a positive diagnostic test among those who actually have cervical cancer relative to the odds of a positive diagnostic test among those who do not have cervical cancer. The SROC is a curve plot with the sensitivity as the y-axis and the false positive rate (i.e., one minus the values of the specificity) as the x-axis.”* |
| Some of the forest plots include just one or two studies. These results should be considered and reported, of course, but I would advise against calling it a “meta-analysis”. I suggest limiting the meta-analysis to situations where there are at least three studies. | | Thank you for taking a careful look at our forest plots. We removed any instance of the term “meta-analysis” in figures that had less than 3 studies. |
| Some of the results are so heterogeneous that a pooled estimate is essentially uninterpretable. In those circumstances it is more useful to explore the reasons for heterogeneity, rather than simply acknowledging its existence. This can be done qualitatively (by trying to understand the difference across studies) or quantitatively (e.g. through meta-regression analysis). Given the relative paucity of the data, a qualitative approach may be more feasible. | | Thank you for this comment. We believe we have given qualitative reasonings for the heterogeneity; we kindly point you to the following sentences in the Discussion section: *“Heterogeneity may be a function of study designs, the dataset, the available data, or a combination of these issues. Further analysis should be done to confirm whether certain variables contribute to increased heterogeneity in outcomes, such as the study region, study size of the population, or capacity of test providers”* (lines 356-360). |
| I must confess I don’t know how to read the pooled SROC curves, especially in Figures 2 and 5. They don’t look like ROC curves in a typical study. I wonder if simply reporting numeric AUC values would be better. | | Thank you for your transparency and careful review of our figures. The AUC value is also provided in the figure for readers who prefer that statistic. |
| It is hard to believe that all studies were of equally high quality (as stated in the discussion). The study quality is really a relative rather than an absolute measure. Surely some studies are stronger than others. For example, only one of the 12 studies was a randomized trial. It would be good to know how its findings compare to those reported in cross sectional studies. | | Thank you for this comment. The quality of the RCT was assessed with the *NIH Study Quality Assessments for Randomized Control Trials* and the observational studies with the *NIH Study Quality Assessments for Observational Studies*. The study team’s senior authors re-assessed the studies and came to the same ratings as the first time they were assessed. |
| One minor point: Some acronyms and abbreviations (e.g. CIN2+, TP, TN, etc.) should be spelled out when they are first mentioned in the text. This is not always the case. | | Thank you. We have made the necessary corrections to make sure that all acronyms and abbreviations are spelled out the first time they are written. |
| *Reviewer #3* | | |
| Some sentences in the results (line 260-263) are repeated. | | The repeated sentence has been removed, thank you. |
| Clear implications of some of the findings e.g DOR for each test is not clear. The study could be more useful to both clinicians and policy makers. How these results are found useful to these groups is not explicit. | | Thank you for this thoughtful comment. We have added the following sentences (see below) and reference (55) regarding recent implications of these results for clinicians and policy-makers. Here are the text: *“Recent World Health Organization guidelines also corroborate our results and conclusions of their use in LMICs; primarily, that policy-makers and governments should be allocating funds and resources for clinician application of primary HPV testing among women.”* |
| The recommendation, Using a point of care HPV screen test lacks clear basis. No costs analysis nor test acceptability comparisons were done in this study. | | Thank you for this comment. We have added three references to support our discussions points regarding the low-cost infrastructure required for HPV point-of-care testing (references 52-54). |
| There is lack of consistency in writing the references. Some follow the standard sentence case, others have predominant capital letters. This needs to be corrected. | | Thank you for your thorough review. We have ensured consistency in the references. |
